# Supplementary material for: The Experience of Post-Stroke Pain and The Impact on Quality of Life: An Integrative Review
Source: Behav Sci (Basel). 2020 Aug 7;10(8):128. doi: 10.3390/bs10080128 (PMC7464541; doi:10.3390/bs10080128)
Supplement: Supplementary file 1 [file behavsci-10-00128-s001.pdf]

Table S1. Demographics.

Study	Methodology, Aim and Eligibility	Participants, Sampling and Setting	Data Collection Methods, Aim and Focus and Analysis
**Choi-Kwon et al., [25]**	*Methodology*: Cross-sectional<br><br>*Aim*: To elucidate the factors that predict the QOL of chronic stage patients with special attention to residual sensory symptoms.<br><br>*Eligibility*: We included only those patients with first-time stroke whose CT/MRI showed lesions compatible with their neurological symptoms.<br><br>Excluded:<br>(1) Strokes due to miscellaneous causes (e.g., aneurysmal rupture, arteriovenous malformation, vasculitis, anticoagulation overuse, or moyamoya disease),<br>(2) TIA<br>(3) Communication problems due to severe aphasia, dementia or dysarthria<br>(4) Previous history of depression that was diagnosed by a medical doctor,<br>(5) Younger than 40 years or older than 80 years of age, since age itself may affect QOL.	*Participants*: *151*<br><br>*Age*: *<63 years*: *73*, *≥63 years*: *78*<br><br>*Gender*: 59 Female, 92 Male<br><br>*Stroke type*: Cerebral infarction: 120, intracerebral haemorrhage: 31<br>*Pain duration*: Not specified<br>*Type of pain*: CPSP<br>*Time post-stroke*: 3 Months and 3 years<br>*Comorbidities*:<br><br>*Sampling*: The subjects included consecutive patients who visited the outpatient clinic of one of the authors (J.S.K.) between 2-5 months post-stroke (mean, 3 months) at the Asan Medical Center between April 1999 and June 2000.<br><br>*Setting* Telephone interview<br><br>*Country*: Korea	*Methods*: Telephone interview<br><br>*Outcome measures*: WHOQOL, VAS, DSM-IV and Barthel Index.<br><br>*Analysis*: The data were analysed using descriptive statistics, Student's t-tests and ANOVA with the SAS statistical package. Parametric statistics were used since the data were normally distributed. Factors that could significantly influence QOL were identified by multiple regression analysis. By employing stepwise regression analysis, all variables were investigated to screen for those factors that were related to changes in QOL. Those factors that did not meet a 0.15 significance level were excluded.
**Galligan et al., [26]**	*Methodology*: Cross-sectional	*Participants*: *98*	*Methods*: Questionnaire

	Aim: The aim of this study was to examine the association of PSF with different aspects of psychological distress, including depression and specific types of anxiety. The relationship between PSF and related conditions such as pain and sleep disruption was also explored.<br><br>*Eligibility*: Over 18 years of age who had experienced a stroke between 1 month and 2 years of data collection.	*Age:* 32-94. Mean: 65.41<br><br>*Gender*: Male 70, Female 28<br><br>*Stroke type*: Ischaemic: 83, haemorrhagic: 6 unidentified: 9<br>*Pain duration*:<br>*Type of pain*: Not specified<br>*Time post-stroke*: 2 years<br>*Comorbidities*: Physical—other CVD: 74, DM: 10, arthritis: 17, other: 27; psychiatric—anxiety: 14, depression: 12, psychosis: 2<br><br>*Sampling*: Participants were recruited to the study from the neurovascular clinic and rehabilitation day hospital of a university teaching hospital and from a local stroke support group.<br><br>*Setting*: Not specified<br><br>*Country*: Ireland	*Outcome Measure*: Numeric pain rating scale (NPRS), Hospital Anxiety and Depression scale, Short Health Anxiety Inventory (SHAI), The Modified Rankin Scale (mRS), Fatigue Assessment Scale (FAS), single-item Likert Scale, Montreal Cognitive Assessment (MoCA) and Stroke-Specific Anxiety Questionnaire.<br><br>*Analysis*: Data were analysed using IBM SPSS Statistics Version 21.0. Relationships between fatigue and psychosocial, demographic and clinical variables were explored using Pearson product moment correlations (for continuous variables) and point-biserial correlations (for dichotomous variables).
**Jonnson et al., [27]**	*Methodology*: Cross-sectional<br><br>*Aim*: To determine prevalence and intensity of pain after stroke, focusing on patients' perspectives<br><br>*Eligibility*: First-time stroke survivors	*Participants*: 297<br><br>*Age*: 17-96. Mean: 72.1<br><br>*Gender*: 119 female, 178 male<br><br>*Sampling*: Lund register<br><br>*Stroke type*:	*Methods*: Quantitative Vignette method<br><br>*Outcome Measures*: VAS, NIH Stroke Scale, Hba1c, Geriatric Depression Scale, Mini-Mental State Examination<br><br>*Analysis*:

		CI 265, ICH 19, SAH 12, UND 1<br>*Pain duration*: Not specified<br>*Type of pain*: Not specified<br>*Time post-stroke*: 4 and 16 months<br>*Comorbidities*: Hypertension: 54%<br>DM: 14%<br>Cardiac disease: 37%<br><br>*Setting*: Outpatient clinic (70%), primary care centres (<10%), nursing homes (<10%) or their own homes (<10%).<br><br>*Country*: Sweden	
**Klit et al., [1]**	*Methodology*: Cross-sectional<br><br>*Aim*: To describe the prevalence and pain types of new onset chronic pain (''novel pain'') in patients with stroke compared with a randomly selected reference group from the general population and to identify factors associated with pain development in stroke patients.<br><br>*Eligibility*: All patients hospitalized with a stroke diagnosis in the County of Aarhus (population 657,671 inhabitants, January 2005) between March 1, 2004 and February 28, 2005 [27] were included in the study.	*Participants*: 608 stroke, 519 reference<br><br>*Age*: Female med age 74.0, male med age 70.9<br><br>*Gender*: 340 male, 268 female<br><br>*Stroke type*: 74 intracerebral haemorrhage, 428 cerebral infarction and 105 unspecified stroke<br>*Pain duration*: Not specified<br>*Type of pain*: Novel pain 39% (237)<br>Headache 10.5% (64)<br>Shoulder 15.1% (91)<br>Other joint 22% (134)<br>Muscle stiffness and spasms 17.4% (106)<br>Other pain 22.9% (139)<br>*Time post-stroke*: 2 years	*Methods*: Quantitative population-based follow-up design and questionnaire.<br><br>*Outcome Measures*: General questionnaire and pain questionnaire.<br><br>*Analysis*:

		Comorbidities: DM 14.1%<br>Depression 20.9%<br>CVD 33.4%<br>*Sampling*: Danish national stroke database and a randomly selected sex- and age-matched reference group from the same catchment area.<br><br>*Setting*: Postal questionnaire<br><br>*Country*: Denmark	
**Kong, Woon and Yang [28]**	*Methodology*: Cross-sectional<br><br>*Aim*: To document the prevalence of chronic pain and to evaluate the effect of pain on quality of life (QOL) in patients 6 months or more after a stroke.<br><br>*Eligibility*: Stroke patients attending the outpatient clinic of a tertiary rehabilitation unit were screened for enrolment into this study and included if post-stroke duration was 6 months or more.	*Participants*: 107 (45 with pain, 62 without)<br><br>*Age*:<br>Age 16-60: 50<br>Age 61-70: 35<br>Age 71-80: 22<br><br>*Gender*: 68 male,<br>39 female<br><br>*Stroke type*: Infarct 68, haemorrhage 39<br>*Pain duration*:<br>*Type of pain*: MSK 32<br>CSPS 13<br>*Time post-stroke*: At least 6 months<br>*Comorbidities*:<br><br>*Sampling*: Outpatient clinic of a tertiary rehabilitation unit	*Methods*: Cross-sectional survey, interviews<br><br>*Outcome Measures*: Patients were interviewed on pain, QOL, mood and functional status by using the short form of the Brief Pain Inventory (BPI), Medical Outcomes Study 36-Item Short-Form Health Survey (SF-36), Beck Depression Inventory and Modified Barthel Index (MBI)<br><br>*Analysis*: The Student's t-test and Mann–Whitney U test were used to analyse continuous variables with normal and non-normal distribution, respectively. The chi-square test was used to examine relations between categorical variables.

		Setting: Outpatient clinic of a rehabilitation centre.<br><br>*Country*: *Singapore*	
**Lindgren, Gard and Brogardh [29]**	*Methodology*:<br><br>*Aim*: To describe experiences of shoulder pain after stroke, how pain affects daily life and perceived effects of interventions.<br><br>*Eligibility*:<br>Daily stroke-related shoulder pain, shoulder pain 4 months post-stroke and<br>decreased sensorimotor function of affected arm, but ability to use to some extent in daily activities	*Participants*: 13<br><br>*Age*: 57-77 (median 65, mean 66.7)<br><br>*Gender*: 6 female, 7 male<br><br>*Stroke type*: Undefined<br>*Pain duration*:5-54 months (median 24 months<br>*Type of pain*: MSK (shoulder)<br>*Time post-stroke*: At least 4 months<br><br>*Sampling*: Not specified<br><br>*Setting*: Interviews took place in rehab unit in quiet room<br><br>*Country*: Sweden	*Methods*: Qualitative interview based on an inductive approach. A semi-structured interview guide was used.<br><br>*Outcome measures*:<br><br>*Duration of interview*: 30-50 minutes<br><br>*Analysis*: Content analysis
**Naess, Lunde and Brogger [30]**	*Methodology*: *Cross-sectional*<br><br>*Aim*: The aim was to evaluate clusters of co-occurring symptoms in the long term.<br><br>*Eligibility*: All consecutive patients with acute cerebral infarction (the	*Participants*: 328<br><br>*Age*: Avg 67.7<br><br>*Gender*: 206 male, 122 female<br><br>*Stroke type*: 328 Cerebral infarction<br>*Pain duration*: At least 6 months	*Methods*: Quantitative self-report questionnaire<br><br>*Outcome Measures*: Questionnaire including a Visual Analogue Pain Scale (VAS), Fatigue Severity Scale (FSS), depression subscale of the Hospital

	index stroke) admitted to the Stroke Unit, Department of Neurology, Haukeland University Hospital, between February 2006 and July 2008, were prospectively registered in a database.	*Type of pain*: Not specified<br>*Time post-stroke*: 6 months<br>*Comorbidities*: Depression 47<br>DM 31<br>Myocardial infarction 38<br>Hypertension 167<br>Smoking 76<br><br>*Sampling*: Admitted to Stroke Unit, Department of Neurology, Haukeland University Hospital<br><br>*Setting*: Postal Questionnaire<br><br>*Country*: Norway	Anxiety and Depression Scale (HADSD) and Barthel Index<br><br>*Analysis*: Logistic regression analysis, linear regression analyses, Pearson's rank correlation (r), 2 test, Fisher's exact test and Student's t-test were performed when appropriate. STATA 11.0 was used for analyses.
**Naess, Lunde and Brogger [31]**	*Methodology*: Cross-sectional<br><br>*Aim*: The aim of this study was to evaluate these symptoms in relation to health-related quality of life (HRQoL) on long-term follow-up.<br><br>*Eligibility*: All consecutive patients with acute cerebral infarction (the index stroke) admitted to the Stroke Unit, Department of Neurology, Haukeland University Hospital, between February 2006 and July 2008, were prospectively registered in a database.	*Participants* 328<br><br>*Age*: Avg 67.7<br><br>*Gender*: 206 male, 122 female<br><br>*Stroke type*: 328 cerebral infarction<br>*Pain duration*: At least 6 months<br>*Type of pain*: Not specified<br>*Time post stroke*: 6 months<br>*Comorbidities*: Depression 47<br>DM 31<br>Myocardial infarction 38<br>Hypertension 167<br>Smoking 76	*Methods*: Questionnaire<br><br>*Outcome measures*: Fatigue Severity Scale (FSS), 14 depression subscale of the Hospital Anxiety and Depression Scale (HADSD),15 EuroQol (EQ-5D), 15D, Barthel Index (BI) and EuroQol Visual Analogue Scale (EQ-VAS).<br><br>*Analysis*: Logistic regression analysis, linear regression analysis, chi-square test, Fisher's exact test, pair-wise correlation test and Student's t-test were performed.

		Sampling: Admitted to Stroke Unit, Department of Neurology, Haukeland University Hospital<br><br>*Setting*: Postal Questionnaire<br><br>*Country*: Norway	
**Sahin-Onat et al. [32]**	*Methodology*: Cross-sectional<br><br>*Aim*: The aim of this study was to assess the effects of central post-stroke pain on quality of life, functionality and depression in stroke.<br><br>*Eligibility*: Excluded other types of pain. Not unconscious or had nociceptive or neuropathic pain due to other causes.	*Participants*: 48<br><br>*Age*: mean age of 60.6±8.5 years<br><br>*Gender*: 28 males, 20 females<br><br>*Stroke type*: Group 1 (with CPSP): 20 ischaemic, 4 haemorrhagic. Group 2 (without CPSP): 18 ischaemic, 6 haemorrhagic.<br>*Pain duration*: Not specified<br>*Type of pain*: CPSP<br>*Time post-stroke*: At least 1 month<br>*Comorbidities*: Yes, but not specified<br><br>*Sampling*: All patients who underwent inpatient rehabilitation treatment at Ankara Physical Medicine and Rehabilitation Education and Research Center were examined between January 2013 and February 2014.<br><br>*Setting*: Ankara Physical Medicine and Rehabilitation Education and Research Center	*Methods*: Twenty-four patients with stroke having central post-stroke pain and 24 similar age- and gender-matched patients with stroke and without central post-stroke pain (Group 2) were enrolled and assessed for effects on quality of life, depression and function.<br><br>*Outcome Measures*: *SF-36*, Functional Independence Measure, Beck Depression Inventory, Visual Analogue Scale, Leeds Assessment of Neuropathic Symptoms and Signs scale.<br><br>*Analysis*: SPSS Statistics 16.0 (SPSS, Chicago, IL, USA) was used for statistical analyses. Descriptive statistics were applied in the analyses. The chi-square test was used to compare the categorical data of both groups. The Mann–Whitney test was applied to compare the continuous variables between two groups. Pearson correlation analysis and linear regression analysis were performed to

		Country: Turkey	identify the relationship between physical and mental scores of the SF-36 QoLS and LANSS score. The level of statistical significance was set at p<0.05.
**Tang et al. [33]**	*Methodology*: Cross-sectional<br><br>*Aim*: The aim of the current study was to examine the associations between PSP and demographic and clinical characteristics, and the effect of PSP on HRQOL in a large sample of stroke survivors at 3 months following their index stroke.<br><br>*Eligibility*: (1) Chinese ethnicity, (2) Cantonese as the primary language, (3) aged 18 years or above and (4) well-documented stroke occurring within 7 days before admission to the study.	*Participants*: 441 (167 with pain, 274 without pain)<br><br>*Age*: With pain: mean 66.1±9.7<br>Without pain: mean 66.1±10.3<br><br>*Gender*: With pain: 88 female, 73 male<br>Without pain: 96 female, 178 male<br><br>*Stroke type*: Not specified<br>*Pain duration*: Not specified<br>*Type of pain*: Not specified<br>*Time post-stroke*: 3 months (Mean 126.5 + 33.5 days)<br>*Comorbidities*: With pain: Diabetes mellitus 59, hyperlipidaemia 81, previous stroke 19, hypertension 125, psychiatric history 13.<br>Without pain: Diabetes mellitus 93, hyperlipidaemia 146, previous stroke 37, hypertension 196, psychiatric history 12.<br><br>*Sampling*: 441 consecutively admitted stroke patients were recruited from the Acute Stroke Unit of the Prince of Wales Hospital (PWH).	*Methods*: Patients (*N* = 441) diagnosed with stroke were assessed with and without pain for multiple outcome measures.<br><br>*Outcome measures*: Modified Rankin Score (MRS), NIHSS, DSM-IV, SF-12, Chinese version of the Faces Pain Rating Scale-Revised (FPS-R). Fatigue Severity Scale (FSS), The Lubben Social Network Scale (LSNS), Barthel Index (BI), Instrumental Activities of Daily Living (IADL), MMSE, Geriatric Depression Scale (GDS) and the anxiety subscale of the Hospital Anxiety and Depression Scale (HADSA)<br><br>*Analysis*: Statistical analyses were performed using the IBM SPSS Statistics, Version 20. Descriptive data are presented as means or proportions, as appropriate. For continuous variables, independent t-tests were performed between the pain and no-pain groups and Chi-square tests were used for categorical variables. A multivariate analysis was used to examine group differences in SF-12 PCS and MCS scores after adjusting for sex,

		Setting: Acute Stroke Unit of the Prince of Wales Hospital (PWH)<br><br>*Country*: Wales	education, depression and BI, GDS, HADSA and FSS scores. Patients' SF-12 PCS and MCS scores were correlated with their AFPS score using Pearson's correlation. The level of significance was set at 0.05 (two-tailed).
**Westerlind et al. [3]**	*Methodology*: Cross-sectional<br><br>*Aim*: To explore the experience of pain 5 years after stroke, and factors associated with the experience of pain.<br><br>*Eligibility*: 18 years old or older at stroke onset	*Participants* 281<br><br>*Age*: 65.4<br><br>*Gender*: 110 female, 171 male<br><br>*Stroke type*: Ischemic 218, haemorrhagic 71<br>*Pain duration*: Not identified<br>*Type of pain*:<br>*76% with pain related disorders*<br>*42% feel in pain*<br>*Time post-stroke*: 5 years<br>*Comorbidities*:<br>36% with musculoskeletal disorder<br><br>*Sampling*: Recruited from Lund stroke in-patients register at Skane Hospital University.<br><br>*Setting*: Data collection at home<br><br>*Country*: Sweden	*Methods*: Quantitative self-report questionnaires<br><br>*Outcome Measures*:<br>Risk Stroke Questionnaire<br>EQ-5D<br>Stroke Impact Scale<br>*Analysis*:<br>Mann–Whitney U test<br>Logistical Regression
**Widar and Ahlstrom [34]**	*Methodology*: Cross-sectional	*Participants*: 43	*Methods*: Quantitative<br>Self-report questionnaires and structured interviews

	Aim: To describe disability after a stroke and how long-term pain influences everyday life according to the Multi-Dimensional Pain Inventory-Swedish language version (MPI-S) and to test the reliability of this instrument.<br><br>*Eligibility*: Patients were identified, 2 years after an acute stroke incident, by means of an inpatient register at a neurological clinic in a hospital in Sweden. Patient selection was based on the Swedish version of ICD-9 and ICD-10, namely infarctus cerebri (433, 434/I63) and haemorrhagia cerebri (431/I61).	*Age*: 30 male, age 33-79, Md 64, 13 female, age 54-82, Md 76<br><br>*Gender*: 13 female, 30 male<br><br>*Stroke type*: Cerebral infarct or haemorrhage (numbers not specified)<br>*Pain duration*: Avg 20 months<br>*Type of pain*: CSPS 15, nociceptive 18, headache 10<br>*Time post-stroke*: 2 years<br>*Comorbidities*:<br>DM 1<br>Cardiac disease 26<br>DM and cardiac disease 8<br>Previously healthy 8<br><br>*Sampling*: Recruited from Lund stroke in-patients register at Skane Hospital University.<br><br>*Setting*: Data collection at home<br><br>*Country*: Sweden	*Outcome measures*: ADL Staircase, Multi-Dimensional Pain Inventory-Swedish language version (MPI-S) and self-reported impairment questionnaire<br><br>*Analysis*:
**Widar, Ahlstrom and Ek, [35]**	*Methodology*: Mixed methods<br><br>*Aim*: To describe HRQOL in persons with long-term pain after a stroke, and to compare this with different types of pain conditions, age, gender and household status.	*Participants* 43<br><br>*Age*: 30 male, age 33-79, Md 64, 13 female, age 54-82, Md 76<br><br>*Gender*: 30 male, 13 female	*Methods*: Qualitative and quantitative methods<br><br>*Outcome measures*: SF-36, Hospital Anxiety and Depression scale<br><br>*Duration of interview*: 30 minutes

	Eligibility: Two years after an acute stroke, patients with cerebral infarcts or haemorrhages, diagnosed by clinical examination and computerized tomography, were identified. The inclusion criteria were an unequivocal stroke episode and long-term pain (>6 months) that began after the stroke.	*Stroke type*: Cerebral infarcts or haemorrhages.<br>*Pain duration*: >6 months<br>*Type of pain*: CSPS 15, nociceptive 18, headache 10<br>*Time post-stroke*: 2 years<br>*Comorbidities*:<br><br>*Sampling*: Recruited from Lund stroke in-patients register at Skane Hospital University.<br><br>*Setting*: Participants' home<br><br>*Country*: Sweden	*Analysis*: Latent content analysis
**Widar, Ek and Ahlstrom, [36]**	*Methodology*:<br><br>*Aim*: To describe pain, coping strategies and experience outcome of coping with long-term pain conditions after a stroke<br><br>*Eligibility*: Cerebral infarct or haemorrhage with pain	*Participants*: 43<br><br>*Age*: 30 male, age 33-79, Md 64, 13 female, age 54-82, Md 76<br><br>*Gender*:13 female, 30 male<br><br>*Stroke type*: Cerebral infarct or haemorrhage<br>*Pain duration*: Avg 20 months<br>*Type of pain*: CSPS 15, nociceptive 18, headache 10<br>*Time post-stroke*: 2 years	*Methods*: Qualitative interview: 2 interviews per participant, 3 months apart, 2 years post-stroke<br><br>*Outcome Measures*:<br><br>*Duration of interview*: No time limitation<br><br>*Analysis*: Latent content analysis

		Sampling: Recruited from Lund stroke in-patients register at Skane Hospital University.<br><br>*Setting*: Conversation in the participants' home environment<br><br>*Country*: Sweden	

Thirteen studies were included. This included 2 qualitative, 1 mixed methods and 10 quantitative-based designs.

Table S2. Modified consolidated criteria for reporting qualitative research tool (COREQ) [23]

**Domain 1: Research team and reflexivity**			**Widar, Ek and Ahlstrom [35]**	**Lindgren, Gard and Bogardh [29]**
Personal Characteristics		***Scoring***		
1	Interviewer/facilitator. Which author/s conducted the interview or focus group?	If they have identified give a point, if they have not give 0, if you are unclear type U.	1<br>The interviews were performed by the first author, Marita Widar.	1<br>Interviews were conducted by the first author, Ingrid Lindgren
2	Experience and training. What experience or training did the researcher have?	Where experience is clearly detailed or a detailed reference to training OR experience is made, a point is given. If unclear or absent, give zero.	0<br>Not mentioned	1<br>Long experience in stroke rehabilitation
Relationship with participants				
3	Relationship established. Was a relationship established prior to study commencement?	Give a point if details of how the researcher or person who undertook qualitative data collection met individuals or if any previous relationship was identified. Where this is unclear, type U. Where this information is absent, type 0.	0<br>Not mentioned	1<br>The interviewer had met some of the participants in a previous study, but had no other relationship with the participants.
4	Participant knowledge of the interviewer. What	A point is scored where information about what the participants knew about the	U<br>An introductory letter with	1<br>They received a letter by mail with

	did the participants know about the researcher? E.g. personal goals and reasons for doing the research	research they were being invited to participate in was mentioned. This includes sending background information and study information sheets. Score zero where this information is absent and U where this information is unclear.	information about the study was sent. Before data collection, the interviewees were provided with written and oral information.	information about the study and were contacted by phone one to two weeks later.
5	Interviewer characteristics. What characteristics were reported about the interviewer/facilitator? E.g. bias, assumptions, reasons and interests in the research topic	Score a point where information about the characteristics of the interviewer and their bias, interests or reasons for participating in research are identified. Score zero where this information is absent. Score U where this information is unclear.	0<br>Not mentioned	U<br>The authors report no conflicts of interest.
	**Total Score for Domain 1.**		**1/5**	**4/5**
	**Domain 2: study design**			
	Theoretical framework			
6	Methodological orientation and theory. What methodological orientation was stated to underpin the study? E.g. grounded theory, discourse analysis, ethnography and phenomenology	Score a point where the paradigm and methodology are given. Score zero where both aspects are missing. Score U where this information is unclear.	0<br>Not mentioned	1<br>A qualitative interview study based on an inductive approach.
7	Non-participation. How many people refused to participate or dropped out? Reasons?	Score a point where the number and the reason or attempts to identify the reason are given (e.g., a point is score if they say participants would not give a reason for non-participation). Score zero where this information is absent and score u where this information is unclear.	1<br>Letter sent to 356 people, 65 declined to participate or did not answer, and 245 responded that they had no pain or that they had other pain conditions. This left a study population of 46; 3	1<br>Twenty-one patients fulfilled inclusion criteria, and eight were excluded for the following reasons: no longer pain (four persons), not able to get in contact with (two persons) and medical reasons (two persons). The final sample included 13 patients.

			dropped out of the study during data collection, leaving 43 participants.	
	Data collection			
8	Interview guide. Were questions, prompts and guides provided by the authors? Were they pilot-tested?	Score a point where testing of the interview script is identified either as a pilot or as a way to determine the content and accuracy of items used. Score a point where consideration to the derivation of questions have come from.	1<br>Interview was in the form of a conversation, without time limitation. During the conversation, the interviewer tried to make sure that the focus was on the pain, which was ascertained by follow-up questions.	1<br>A semi-structured interview guide, developed by the authors, was used.
9	Field notes—Were field notes or reflective diary made during and/or after the interview or focus group?	Score a point where field notes are identified. Score zero where field notes are not identified. Score U where this information is unclear.	0<br>No mention of field notes or reflective diary.	0<br>No mention of field notes or reflective diary.
10	Data saturation. Was data saturation discussed?	Score a point where saturation of data is considered. Score a point where another form of sample size reference is made. Score U where this information is unclear.	0<br>Not mentioned.	0<br>Not mentioned.
		**Total for Domain 2**	**2/5**	**3/5**
		**Domain 3: analysis and findings**		
	Data analysis			
11	Description of the coding tree. Did authors provide a description of the coding tree?	Score a point if an audit trail is given. Score a point if a coding tree is mentioned or score a point if another technique is mentioned that provides a way to structure the information gained.	1<br>Meaning units were identified. Themes were formed.	1<br>From the transcribed interviews, meaning units answering the study aims were identified from the data. The meaning units were then labelled with codes. The codes were then sorted into subcategories as well as categories to answer the study aims.

12	Derivation of themes. Were themes identified in advance or derived from the data?	Score a point if data-driven or theory-driven coding is identified or if it is clear how analysis was determined.	1<br>Themes, categories and subcategories were identified and derived from data.	1<br>The analysis resulted in three categories and seven subcategories, derived from data.
	Reporting			
13	Clarity of minor themes. Was there a description of diverse cases or discussion of minor themes?	Score a point if there is sub-detail for each major theme. Score no points where this information is absent or U if this information is unclear.	1<br>Subcategories were identified.	1<br>Seven subcategories.
	**Total for domain 3**		3/3	3/3
	**Grand total**		7/13	10/13

Table S3. Cochrane's risk of bias tool [21].

**Study**	**1**	**2**	**3**	**4**	**5**	**6**	**Summary within Trial**	**Comments on High Risk Components**	**Overall quality**
Choi-Kwon et al., [25]						H	3	Other (Observer Bias): Telephone interview<br><br>Other (Sampling Bias): Not representative of the population as cognitive, speech and severely disabled who were unable to attend out-patients clinic were excluded.<br><br>Other (Maturation Bias): Between 3 months and 3 years, other factors may influence results.	High risk
Galligan et al., [26]			H			H	2	Other (Sampling Bias): Not representative of the population. The sample comprised individuals without significant cognitive, physical or communicative impairments.	Moderately high risk

				Detection Bias: Comorbidities make it difficult to differentiate the influence of pre-stroke, post-stroke and non-stroke factors on domains.	
Jonsson et al., [27]		H	1	Sampling Bias: Participants were assessed with VAS scores on both follow-ups and were excluded if they were over 79.7 or had more severe stroke, or both. This means participants have low NIHSS scores and a low mean age compared with other studies and not representative of a stroke population.	Moderately low risk
Klit et al., [1]		H	1	Other (Recall Bias): Retrospective study.	Moderately low risk
Kong, Woon and Yang [28]		H	2	Other (Sampling Bias): Not representative of the stroke population—excluded significant aphasia and cognitive impairment. It also examined patients in an outpatient clinic; they were preselected for having good rehabilitation potential. The small number of patients in nursing homes and the generally high MBI scores reflect this bias.<br>Other (Measurement Error): BPI has not been validated as an outcome measure in the stroke population.	Moderately high risk
Naess, Lunde and Brogger [30]		H	1	Other—Sampling Bias: Not representative of the population. Responders had smaller strokes than the non-responders so reported frequencies of pain, fatigue and depression are likely lower than if people with larger strokes had responded. This may also mean a higher HRQOL.	Moderately low risk
Naess, Lunde and Brogger [31]	H	H	2	Other—Sampling Bias: Not representative of the population. Responders had smaller strokes than the non-responders so reported frequencies of pain, fatigue and depression are likely lower than if people with larger strokes had responded. This may also mean a higher HRQOL.<br>Attrition Bias: Response rate 61% and complete data obtained from 55%.	Moderately high risk

Sahin-Onat et al., [32]		H	1	Other (Sampling Bias): Not representative of stroke population because all patients could speak and cognitively understand the tests.<br><br>Other (Sampling Bias): Small sample size of 24.	Moderately low risk
Tang et al., [33]		H	1	Other (Sampling Bias): Not representative of stroke population because it excluded cognitive impairment and participants with aphasia.	Moderately low risk
Westerlind et al., [3]	H			Attrition Bias: Response rate 61%. Non-responders were significantly more women (p = 0.001)	Moderately low risk
Widar and Ahlstrom [34]	H	H	2	Sampling Bias: Not representative of stroke population because it excluded speech difficulties.<br><br>Attrition Bias: Internal dropout.	Moderately high risk
Widar, Ahlstrom and Ek [35]	H	H	2	Attrition Bias: Incomplete outcome data. Of the total number of participants included in this study (N = 43), the qualitative interview was not performed for one participant as they were not inclined, and one other had missing recorded data due to issues with the tape recorder. There were two dropouts on SF-36 because participants did not want to answer the questionnaires, and one was excluded because of incomplete answers. There were two dropouts for the HAD Scale, the same participants as in SF-36. There was an internal dropout on the anxiety subscale by one respondent on five items.<br><br>Other (Sampling): Not representative of stroke population because of exclusion of patients that could not communicate and those with other pain conditions.	Moderately high risk

Risk of bias criteria: 1: Selection Bias. 2: Performance Bias. 3: Detection Bias. 4: Attrition Bias. 5: Reporting Bias. 6: Other. Levels of risk of bias: H, high risk of bias; U, unclear risk of bias; L, low risk of bias.

Table S4. Criteria for risk of bias.

Risk of Bias	Criteria	Conclusion

Low risk	Study avoids bias.	High quality. Therefore, findings can be taken with limited caution.
Moderately low risk	1 risk of bias.	Remain optimistic about findings.
Moderately high risk	2 risks of bias. Study has potential for some bias.	Findings should be interpreted with caution.
High risk	3 risks of bias. Study has flaws in design and/or execution that increase potential for bias that may invalidate study results.	Poor quality and study has significant potential for bias; therefore, findings cannot permit a conclusion. Requires more research of high-quality studies to confirm findings.

Summary

4x Moderately Low, 5x Moderately High, 1x High.

Table S5. Summary of outcome measures used in quantitative studies.

	Outcome Measures							
**Study**	**QOL**	**Pain**	**Depression**	**Anxiety**	**Performance in ADL/Function**	**Fatigue**	**Cognitive Deficit**	**Other**
Choi-Kwon et al [25]	WHOQOL	Visual Analogue Scale (VAS)	Diagnostic and Statistical Manual of Mental Disorders, fourth edition (DSM-IV)		Barthel Index (BI)			
Galligan et al [26]		Numeric Pain Rating Scale (NPRS)	Hospital Anxiety and Depression Scale (HADS)	Short Health Anxiety Inventory (SHAI)	The Modified Rankin scale (MRS)	Fatigue Assessment Scale (FAS)	Montreal Cognitive Assessment (MoCA)	

				Stroke-Specific Anxiety Questionnaire		Single-item Likert Scale		
Jonnson et al [27]	Interview questions	Visual Analogue Scale (VAS)	Geriatric Depression Scale (GDS)		NIH Stroke Scale (NIHSS)		Mini-Mental State Examination (MMSE)	Hba1c
Kilt et al [1]	General quality of life questionnaire	Pain Questionnaire	Questionnaire					
Kong, Woon and Yang [28]	36-Item Short-Form Health Survey (SF-36)	Brief Pain Inventory (BPI)	Beck Depression Inventory (BDI)		Modified Barthel Index (MBI)			Sensory deficits
Naess, Lunde and Brogger [30]	Questionnaire	Visual Analogue Scale (VAS)	Depression subscale of the Hospital Anxiety and Depression Scale (HADSD)		Barthel Index (BI)	Fatigue Severity Scale (FSS),		
Naess, Lunde and Brogger [31]	EuroQol (EQ-5D), 15D, EuroQol Visual Analogue Scale (EQ-VAS)	EQ-VAS	Depression subscale of the Hospital Anxiety and Depression Scale (HADSD)		Barthel Index (BI)	Fatigue Severity Scale (FSS)		
Sahin-Onat et al [32]	36-Item Short-Form Health Survey (SF-36)	Visual Analogue Scale (VAS)<br><br>Leeds Assessment of Neuropathic	Beck Depression Inventory (BDI)		Functional Independence Measure (FIM)			

		Symptoms and Signs scale (LANSS)						
Tang et al [33]	SF-12—Physical component summary (PCS) and mental component summary (MCS)	Chinese version of the Faces Pain Rating Scale-Revised (FPS-R).	Diagnostic and Statistical Manual of Mental Disorders, fourth edition (DSM-IV)<br><br>Geriatric Depression Scale (GDS)	The anxiety subscale of the Hospital Anxiety and Depression (HADSA)	The Modified Rankin scale (MRS)<br><br>NIH Stroke Scale (NIHSS)<br><br>Barthel Index (BI)<br><br>Instrumental Activities of Daily Living (IADL)	Fatigue Severity Scale (FSS)	Mini-Mental State Examination (MMSE)	The Lubben Social Network Scale (LSNS)
Westerlind et al [3]	EQ-5D<br>Stroke Impact Scale	Risk Stroke Questionnaire						
Widar and Ahlstrom [34]	Multidimensional Self-Reported Impairment Questionnaire	Pain Inventory-Swedish language version (MPI-S)						
Widar, Ahlstrom and Ek, [35]	36-Item Short-Form Health Survey (SF-36)	Interview	Hospital Anxiety and Depression Scale (HADS)					

Table S6. Summary of quantitative evidence.

Domain	Outcome Measure	Study	PSP Main Findings	QOL Main Findings

Anxiety	The anxiety subscale of the Hospital Anxiety and Depression (HADS-A)	Tang et al [33]	Pain was significantly associated with anxiety ($p = 0.003$).	
	Short Health Anxiety Inventory (SHAI)<br><br>Stroke-Specific Anxiety Questionnaire	Galligan et al [26]	Pain was significantly and positively associated with health anxiety (no statistical or numerical change provided).	
Depression	Hospital Anxiety and Depression Scale (HADS)	Galligan et al [26]	No association.	
		Widar, Ahlstrom and Ek [35]	Pain type (CPSP vs nociceptive vs headache) was found to have no association.	
	Depression subscale of the Hospital Anxiety and Depression Scale (HADSD)	Naess, Lunde and Brogger, [30]	No association.	
		Naess, Lunde and Brogger, [31]		Significant and negative association (r = -0.50) between depression and HRQOL ($p < 0.001$), as measured by the 15D, but no association on the EQ-5D.
	Beck Depression Inventory (BDI)	Sahin-Onat et al [32]	No association.<br>MCID, difference of 28% on the BDI.	
		Kong, Woon and Yang [28]	No association.	Kong et al. (2004) found that depression had a negative influence on five out of eight domains of the SF-36. Depression was significantly associated with general health ($p < 0.01$), vitality ($p < 0.01$), social functioning ($p < 0.01$), role limitations due to emotional problems ($p = 0.02$) and mental health ($p < 0.01$).

	Geriatric Depression Scale (GDS)	Jonnson et al [27]	Significant positive association (coefficient B = 2.1) between higher pain intensity and depression ($p < 0.001$).<br>Younger age associated with higher pain intensity ($p = 0.006$).	
		Tang et al [33]	Significant association between pain and depression ($p = 0.009$)	
	DSM-IV	Tang et al [33]	Significant association between pain and depression ($p = 0.081$)	
		Choi-Kwon et al [25]		Depression at 3 months post-stroke was significantly associated with a low QOL at 3 years post-stroke ($p < 0.01$).<br>A very small effect size was calculated among patients without depression (Hedges' $g = (3.49 – 3.45)/0.47 = 0.085106$). A small to medium effect size was calculated among patients with depression (Hedges' $g = (2.89 – 2.97)/0.21 = 0.380952$).
	Questionnaire	Klit et al [1]	Significant association between pain and depression ($p < 0.001$, CI: 3.43 (2.25–5.25)). A positive association between pain and low age (<65 years) ($p < 0.001$, odds ratio: 2.45 (1.71-4.45).	
		Westerlind et al [3]	Participants who reported having more frequent pain reported experiencing depression ($p < 0.001$).	
Fatigue	Fatigue Assessment Scale (FAS)<br><br>Single-item Likert Scale (sleep disturbance)	Galligan et al [26]	Significant positive association (r = 0.21) between fatigue and sleep disturbances ($p < 0.05$).	

		Naess, Lunde and Brogger [30]	Significant and positive association (r = 0.27) between pain and fatigue ($p < 0.001$).	
	Fatigue Severity Scale (FSS)	Naess, Lunde and Brogger [31]	Significant and negative association (r = -0.44) between pain (as measured by EQ-VAS) and fatigue ($p < 0.001$).	No association between fatigue and QOL (EQ-5D). Significant negative association (r = -0.34) fatigue and QOL (15D) ($p < 0.001$). Significant negative association (r = -0.37) between sleep disturbances and QOL (15D) ($p < 0.001$).
		Tang et al [33]	Significant and positive association between pain and fatigue ($p < 0.001$)	
Cognitive Function	Montreal Cognitive Assessment (MoCA)	Galligan et al [26]	Pain caused catastrophising, hypervigilance and biased attention to somatic symptoms (no statistical or numerical change provided).	
	Mini-Mental State Examination (MMSE)	Tang et al., 2015 [33]	Significant association between pain intensity and increased cognitive decline (a lower MMSE score) (p = 0.038).	Significant negative association (r = -0.145) between pain and MCS ($p < 0.071$).
		Jonnson et al [27]	Significant positive association between pain intensity and less cognitive decline (a higher MMSE score) (p = 0.004).	
Physical Function	Barthel Index (BI)	Choi-Kwon et al. [25]		Being dependent in ADLs was significantly and negatively associated with QOL ($p<0.01$). A small effect size was calculated among patients with a BI <96 (Hedges' g = (3.16 - 3.05)/0.485207 = 0.226707). A very small to small effect size was calculated amongst patients with a BI ≥96 (Hedges' g = (3.51 - 3.58)/0.436494 = 0.160369). A significant negative association between motor dysfunction and QOL ($p<0.01$).

	Naess, Lunde and Brogger, [30]	Significant negative association (r=-0.33) between the number of symptoms and the Barthel Index ($p<0.001$).	
	Naess, Lunde and Brogger, [31]		Significant positive association (r=0.55) between the BI and QOL, as measured by the EQ5D ($p<0.001$).
	Tang et al [33]	No association.	
Modified Barthel Index (MBI)	Kong, Woon and Yang [28]	No association between pain and physical function.<br>MCID difference of 2.7 points.	
Modified Rankin Scale (MRS)	Galligan et al [26]	Fatigue significantly associated (r=0,244) with functional impairment ($p<0.05$).	
	Tang et al [10]	No association.	
NIH Stroke Scale (NIHSS)	Jonnson et al [27]	Significant association between a higher NIHSS score (decreased function) and pain intensity ($p<0.001$).<br>Females have more impaired functional status (lower BI) ($p=0.038$) and a higher pain intensity ($p=0.006$).	
	Tang et al [33]	No association.	
Functional Independence Measure (FIM)	Sahin-Onat et al [32]	No association between pain and physical function. Severity of pain decreased with activity. Neuropathic Symptoms and Signs scale (LANSS) and physical score of the SF-36 ($p=0.0001$).	Significant negative association was found between pain and physical role limitation ($p<0.05$) and physical score ($p<0.01$) domains of the SF-36.

	Leeds Assessment of Neuropathic Symptoms and Signs scale (LANSS)	Sahin-Onat et al [32]	Significant negative association (r=-0.619) between the Leeds Assessment of Neuropathic Symptoms and Signs Scale (LANSS) and physical score of the SF-36 (p=0.0001).	
	Instrumental Activities of Daily Living (IADL)	Tang et al [33]	No association.	
	SF-12	Tang et al [33]		Significant negative association (r = -0.260) between pain and PCS (p<0.001).

Table S7. Summary of qualitative evidence.

Study	Results	Comments HP
**Lindgren, Gard and Brogardh [29]**<br>Shoulder pain after stroke – experiences, consequences in daily life and effects of interventions: a qualitative study	**Multiple pain characteristics.**<br>**Insidious pain onset and varied pain descriptions.**<br>Most of the participants described the pain onset as insidious and had difficulties to precisely describe when the pain had started. It just started aching a little, then it grew more and more (P13)	Onset of pain - gradual<br>Onset of pain - unknown start
	Dull ache was described by most participants. It's grinding… yeah more a rough grinding, hurts, oh, but it's not exactly as a quick stabbing pain (P4), but some described the pain as a knife stab or short pain …kind of like as if you've stuck a knife straight into the elbow and shoulder (P8)). Pain as a different sensation, as stitching or burning, was also described, …it's also burning, sometimes it feels like as if I had pain in the shoulder and then it's just as if the blood was to stop here by the elbow and sometimes it goes down the entire arm and hand (P13) …as if the skin's too small, it tightens really hard (P5).	Progression of pain – aching grew over time<br>Type of pain – dull ache/grinding/ stabbing/stitching/burning<br>Progression of pain – from shoulder down to hand<br>Location of pain
	**Pain varied over time and in situations.**<br>The pain varied widely over time among the participants. While some participants described that the pain had gradually decreased since the onset, others had constant pain. Now I've almost, I've gotten rid of that	Progression of pain – decreased/constant/increased

	pain, it doesn’t hurt anymore (P11). It hasn’t gotten worse but it hasn’t gotten better either. It’s just constant all the time… (P10). For some participants, the pain had increased over time …well I can say it’s gotten worse, it’s gotten worse and worse and worse (P1).	Positive thoughts
	A great pain variation during day and night, or over days or weeks, was also described. Most participants described pain during waking hours. For some participants, pain could be worse in the morning and for others, in the afternoon or evening. They [the stinging pain sensations] are the worst in the afternoons, from 2 PM until… well, I suppose… until dinner time in the evening (P9). Some participants experienced pain for a short while or up to a few hours after unsuitable movements or activities. I only have touches [from the pain] sometimes when I get my arm in a wrong position… maybe for half an hour [the pain lingers] (P11). More than one third described pain during the night. Five nights a week or so [I have pain in my shoulder], so that’s almost every night (P13). Others experienced worse pain over periods of several weeks …it can be several, yeah several weeks sometimes (P1). A majority of the participants described pain during movements; when lifting the arm too high (over the horizontal plane) or moving the arm behind the back, for example, when putting on a shirt …when I do those movements like undressing or dressing or lifting the arm (P3), after exercise or activities with high load or long duration …these pains emerged the more I moved and strained this arm (P8). On the other hand, some participants experienced more pain at rest …it [the pain] becomes more pronounced when I don’t activate my brain (P5). Laying down on the paretic side or in situations when the arm hung down by one’s side could also cause pain … if the arm is hanging down or laying by the body, I have to lift it and put in on my stomach, then it eases the pain (P11), but one participant described the opposite and reflected on the inconsistency …it’s better [to lie on the aching side]… which is weird because there’s pressure on it (P13). Aggravating factors such as a generally high muscle tension, cold or warm weather or upper respiratory infection were described to increase the pain by some participants.	24 h pattern of pain<br>Type of pain - stinging<br>Duration of pain<br>Pain-inducing movements<br>24 h pattern of pain<br>Duration of pain<br>Pain-inducing movements<br>Pain-reducing movements<br>Aggravating factors of pain – physiological/environmental - weather/ illness

		Progression of pain – illness increased pain
	Lack of explanation to the pain.<br>Several participants reported that they had not received any explanation for the pain, while others did…no it's a blank slate [what causes the pain] (P6) … they said that it [the pain] doesn't disappear until you die, they said (P10). One participant would like to have an explanation …yeah [I've missed] some explanations of what causes it. I understand that not all axons work but how are they… is it gonna continue like this forever or what's gonna happen? (P12). One participant was satisfied without any explanation …it was enough for me to note that it [the pain] was connected to the stroke (P8), and another participant had found a personal possible explanation …neuropathic pain, nobody has used that word until I started using it myself (P9).	Incomprehensibility - lack of understanding of cause<br><br>Want to know prognosis
	Activity and social limitations in daily life. While some participants experienced that the pain did not hinder activities… I screw screws, I do everything (P5), others described that they had to adjust activities to the pain or accept the pain …if you're gonna do something… it's not fun, you don't have any energy to do anything because it hurts so bad …today I have to refrain (P1) Sometimes it can hurt really bad when you use your hand in the wrong way …like in gardening (P6). Often both pain and decreased sensorimotor functions in the arm and hand contributed to the perceived activity limitations. Even if the participants were independent in personal care, dressing took a long time because of difficulty in using the arm and hand. What previously took 10 minutes to put on, now takes over an hour (P10). Pain while cooking and eating was described …now I've stopped cooking because I can't do it (P10). Some participants had given up leisure activities …I was very active before… playing golf and tennis, and I can't do that now (P2). Some participants felt that the pain did not affect social situations and that social events even led to less pain …you're so occupied by other things that you don't think about it [the pain] (P11). Others refrained from	Positive thoughts<br><br>Adaptation of activities<br>Acceptance of the pain<br><br>Pain-inducing movements<br>Cause of limitation<br><br>Adaptation of activities – to accommodate taking longer<br><br>Physical decline<br>Unable to do certain activities

	socializing with others or felt that they sometimes got tired and had to withdraw for a while. Definitely, you don’t have the energy to go out and things like that, eating dinner and stuff, it’s all over with (P4).	Distraction reduces pain – coping mechanism
	Emotional reactions. About half of the participants expressed emotional reactions because of the pain. Some days you pity yourself a little extra. And when your arm aches… you think… you hope you fall down the stairs and break your neck (P10). Many nights I’ve cried because I’ve had such pain. And I’m not usually one to cry (P 13), while others perceived less problems or described that the pain hindered to a lesser extent compared to other stroke-related problems …well, the pain hinders me to… it’s like around 30, 40% I’d say [of all consequences after stroke] (P2). Some participants experienced that the shoulder pain led to tiredness or fatigue, but that it was difficult to sort out which role the pain played. If the fatigue is connected to the stroke or that I have pain, I have a hard time estimating what causes that fatigue (P5). Pain during the night had a great impact on some participants,<br>…you go to bed [on the aching side] and you think – oh, nothing. But then you wake up in pain because it’s as if the entire arm is numbing (P10) I don’t like going to bed. I think “Oh now I have to go to bed, now I’ll feel pain” (P13). Pain could affect mood and some participants expressed anger at the pain I almost got angry. This pain was of such caliber that it like affected me negatively (P8). A feeling of depression was also described, either that the pain led to a depressive feeling or that a depressive mood increased the pain,<br>…how you’re feeling mentally, eh if everything’s okay you’re not hurting as much… but when you’re down in the dumps everything seems very, very heavy and it aches (P1) Yeah it [the pain] has affected me. I’m not as happy or, you know (P13).	Fatigue<br>Negative thoughts - pain can heighten emotions<br>Fatigue<br>Incomprehensibility<br>24 h pattern of pain<br>Type of pain - numbing<br>Disrupts sleep<br>Mood – anger/depression
	A variety of pain interventions and their effects. Gentle arm exercises were described by most participants, but also more general methods such as exercises in a warm pool or walks. A few participants had tried	Progression of pain – depressed mood increased pain

	cold treatment, massage, corticoid injections, strength exercises, strapping, orthosis, transcutaneous electrical stimulation or acupuncture. Pain medication was used by less than half of the participants. The gentle arm exercises that relieved the pain were often initiated by healthcare professionals. A few participants received help with movement exercises, while others managed to perform them on their own. Participants described that their own responsibility and continuity was important. They were also proud that they could handle the pain by themselves, I have some exercises against walls… and three to four other exercises like that for the hand and up and down and all around (P7) …I think this arm exercises thing has helped me the best (P13) …it's a lot about this tediousness, for example holding your palms against each other, moving them forward and then angling them upwards, everything slowly and then, you don't actually do the movements with the help of the muscles, but you do them with the help of your mind (P8). Exercises in a warm pool and walks were examples of more general interventions initiated by professionals which some participants experienced as pain relieving. A lot of water exercise, because I discovered… that it helped me. Helped me a lot. So I've stuck to that and although especially new arm exercises hurt, I've stuck to it (P12) I've been to a physiotherapist… she claims loud and clear… if you concentrate on walking and exercising this [the pain] will come to be in second hand …I [have to] admit that the more I walk, the less I feel the pain (P5). A few participants had tried other interventions on their own, for example, liniments, cold treatment or warm showers, which were perceived as effective. It [showering in hot water] lowers a lot, if I'm hurting in the morning and to step into the shower feels REALLY GOOD afterwards (P13). Meditation was also used to master the pain. Massage was also tried, but, without any perceived effect. Some participants reported that botulinum toxin injections in the shoulder had been discussed, but had never been realized. Moreover, the single participants that had been recommended corticoid injections, strength exercises, strapping, orthosis,	Type of pain – heavy, aches<br><br>Pain-reducing movements<br><br>Interventions to ease pain<br><br>Handling pain independently<br>Self-worth/strength/ gives patient control/ self-management<br><br>Interventions to ease pain

	transcutaneous electrical stimulation and acupuncture had bad experiences of these interventions …then I've had this…orthosis… but it didn't feel good… I felt as if I had a straitjacket on me… that was tough (P11).<br><br>Cautiousness to pain medication. Some participants used medication such as NSAID, simple or prescribed pain medication such as paracetamol or prescribed opioids …yeah, if I'm hurting I put some pain cream on or take a tablet (P11). Tiredness as a side effect of pain medication was also described. However, several participants expressed a restrained attitude to pain medication. The causes mentioned were a fear of taking too many or too many types of medication or a fear of not being able to drive …I drive a lot so I don't want to be restricted in my own perception of things that I see as a lot more important (P5).	24 h pattern of pain<br>Failed interventions to ease pain<br><br>Side effects – tiredness/altered perceptions<br>Fear of polypharmacy
**Widar, Ek and Ahlstrom, [36]**<br>Coping with long-term pain after stroke	Pain Description (Table 1). All the participants described where their pain began, its extension over time and where they had the most pain. There was a great deal of variation in the narratives regarding pain intensity among the participants. Most participants used pain descriptors such as unpleasant, troublesome, annoying and tiring. The pain occurred at any time, but also on specific occasions that are described under the specific pain types.<br><br>CSPS—Participants suffering from central post-stroke pain (central pain) described pain in the entire half of the body, or in an arm, a leg, or just a hand or foot. The pain was continuous or was felt at least for a few hours daily. In addition to the sensation of burning, the participants also experienced pain as cutting, dull and/or numbness. The pain occurred in	Description of pain<br><br>Location of pain<br>Duration of pain<br>Type of pain – burning, cutting, dull, numbness, gnawing, cramping

	connection with static body positions or in connection with certain physical motions. Often pain was associated with or involved touch, i.e., when the participants touched something or they were touched by someone else. Cold, windy conditions and temperate water were also reported as provoking pain.	Pain-inducing movements<br><br>Aggravating factors – environmental – weather and touch
	Nociceptive—Participants suffering from nociceptive pain described pain in the affected shoulder/arm or leg, some with radiation to various parts of the arm and hand. Most common was an extended localization, with pain increasing or decreasing overtime. The pain was usually continuous and was worst in the evening. It was described as ranging from endurable to violent and was also experienced as dull, gnawing or cramping. The pain most commonly occurred when the shoulder/arm was under physical exertion or load. Lying on the stroke-affected sore side of the body or even sitting still could exacerbate the pain.	Progression of pain<br><br>Duration of pain<br>24 h pattern<br><br>Aggravating factors – physical exertion or load<br>Aggravating factors - position
	Headache—Participants suffering from tension-type headache had a different kind of headache after their stroke than what they had experienced previously, and they could clearly specify the localization of the headache. They reported that the headache would come at intervals, usually two to three times a day, or a few times each week or month. The headache might be present in the morning upon awakening and/or occur in the afternoon in connection with tiredness. Headache was described as ranging from dull to terrible. Other experiences included a sense of heaviness in the head or stabbing, but primarily the headache was worrying and terrifying. Physical exertion sometimes provoked a headache, but mainly it was provoked by stress associated with time pressure or involvement with too many activities.	Duration of pain<br>24 h pattern<br>Fatigue<br>Type of pain – dull, heavy, stabbing<br>Emotion/mood associated with pain<br>Aggravating factors – physical exertion<br>Aggravating factors – stress
	Incomprehensibility emerged in those participants lacking clear knowledge and understanding of the relationship between their stroke and the pain, of the causes of their pain, or of the extent to which it would disappear or get worse or whether any treatment was available.	Incomprehensibility - lack of understanding of cause

	Those with tension-type headache expressed their anxieties in different ways, wondering whether there might be something wrong inside their heads.	Want to know prognosis<br>Negative thoughts
	Disturbed sleep was a major problem among participants with central or nociceptive pain, due to the inability to find a comfortable body position in bed. While participants experiencing nociceptive pain awoke one or more times each night, a few participants with central pain did so when they felt the bed too hot.	Aggravating factors – inability to find comfortable sleeping position disturbs sleep<br>24 h pattern
	Fatigue was manifested in the descriptions, mainly among participants with continuous pain and with tension-type headache. They described having insufficient energy for planned activities since they experienced their pain as exhausting. Participants with nociceptive pain spoke specifically about feeling tired in their arms, and about being tired before the evening. Among participants with tension-type headache, fatigue constituted a major problem since they never felt rested.	Fatigue
	Diminished capacity in daily life, not only physical but also mental, emerged in the descriptions from participants with central or nociceptive pain. They described major impediments because of their pain in their daily activities, personal care and hobbies. It took longer to do everything, primarily among those who were forced, due to pain in their other arm/hand, to use the arm/hand they usually did not work with. On the other hand, those with tension-type headache experienced difficulty in watching television or reading for extended periods of time. The following quotation illustrates diminished capacity as described by a previously very active person: "I have to give up a lot. I used to love to dance. I can still do it, but I have to take it unbelievably easy. It really hurts in my leg when I do it."	Physical decline<br>Adaptation of activities<br>Physical decline
	Mood changes were described as involving a depressed frame of mind. The pain was more precisely described as disheartening, enervating and	Mood – depression/anger

	worrying and the participants found themselves to be more irascible or angry than usual. Participants with nociceptive pain expressed a desire to “get rid of the arm.” The following quote illustrates the impact of pain on mood: “Both things are equally troublesome, the motor impairment and the pain. The best thing you can imagine would be to sleep 15 hours a day. Then the hours when you were awake, you would be pain free and feel happy. Yes, no pain then. When you find yourself in this situation, it’s pretty hard to see the bright side.” Distress due to fear or anxiety concerning whether a new clot was forming was particularly prominent among those with tension-type headache, even though they did not have a headache in connection with the stroke incident. “But I think that what is strange is that this can happen inside my head at any time, and I get scared. I think that something is going to happen to me, and now it’s starting again.”	Negative thoughts<br><br>Incomprehensibility/mood
	Stress in relationships involving family and married life, friends, and caregivers occurred because of pain. One reason for this was that many, alone or together with their family, participated less often in various physical and/ or social activities they had participated in before. This was a source of increased stress for their spouses/families. They also had the perception that caregivers, and in some cases relatives, did not have enough time for them. In addition, they also described situations in which they did not want to show that they were in pain.	Reduced social activities<br>Stress in relationships - patients worried about causing stress to their relations.<br>Lack of social support - felt they were not given enough time by their relations
	Coping Strategies<br>Making the Pain Comprehensible (Table 1). The participants described what they imagined to be the causes of their pain. They did not know whether the problems were attributable to overexertion, antihypertensive medication, newly prescribed or long-term treatment or age, or whether they were a punishment. Participants with central pain often came to the conclusion that the problem was due to nerves in the painful region. Among those with tension-type headache, a few	Incomprehensibility - lack of understanding about the cause of pain<br>Aggravating factor – overexertion

imagined the possibility that the infarct was still present and creating pressure in the head.	
Efforts to Avoid and to Relieve Pain (Table 1). Planning various physical activities over time was done mainly among participants with central or nociceptive pain, as an effort to avoid pain. In order to avoid overexertion in relation to daily activities, they sometimes planned activities together with their relatives, mainly more extensive cleaning and grocery shopping. Those who were working worked shorter hours because of tactile allodynia, physically heavy labor or stress. Participants with nociceptive pain also described other sorts of planning. They said that they avoided rapid movements or specific arm positions and used the pain-free arm or both hands for certain tasks. The participants described various solutions in order to avoid pain, mainly in connection with carrying, lifting or putting on clothes: "When I put on a t-shirt over my head, I always put the left hand in first, and if I'm standing up and putting on my shoes, I use my right hand rather than my left, since it hurts if I let my arm hang forward and downward." Avoidance of mentally stressful activities was cited most notably by participants who experienced tension-type headache. Efforts to avoid such activities involved taking protective measures at work to guard against disturbing noises. Furthermore, there were planning and avoidance of activities requiring concentration, such as reading or doing crossword puzzles, and resting: "I have to think about avoiding putting myself into situations where I will become stressed. You also have to bear in mind that you're tired, and that you have to go and lie down and take an afternoon nap or rest before you go out."	Strategy to avoid pain - pacing of activities to manage fatigue<br><br>Strategy to avoid pain - asking for help to avoid overexertion (agg factor)<br>Mood – stress leading to reduced working hours<br>Strategy – working shorter hours<br><br>Adapt - avoid aggravating movements/use the other arm<br><br>Avoidance behaviours – noises/concentration/stressful situations
Changing body position was reported by participants with central or nociceptive pain, who tried, both in the day and at night, to eliminate or alleviate their pain. They often turned over or got up and moved when lying down or sitting: "I change positions many, many times during the night, and I feel the very best when I'm moving." Participants with	Fatigue

	motor impairment reported that they would attempt to move the body part to the extent that they were able to. This also entailed, among participants with nociceptive pain when they were in pain, using the other arm/hand or both for various activities.	Pain-reducing movements
	Physical exercise was reported by participants with nociceptive pain in order to prevent pain by stretching their shoulder/arm using training equipment, or aquatic exercise. In order to relieve their pain, participants with central pain did perform physical stretching exercises, but were careful to avoid overexertion. The participants with tension-type headache reported that they went out for a walk to get some fresh air in an attempt to relieve the headache.	Adapting<br>Aggravating factor – overexertion
	Massage was reported by those with central pain and with limited extension of their pain, while a few participants had gone to masseurs.	
	Participants with nociceptive pain used transcutaneous electrical nerve stimulation (TENS).	
	Taking medications in order to avoid pain included a few participants who took sleeping aids or medications before engaging in social activities. They reported doing this so that they could move more freely and sit for a longer time. The use of medications, mainly non-prescription analgesics such as paracetamol (acetaminophen), acetyl acid or ibuprofen, was common. These were sometimes taken by participants with central or nociceptive pain in combination with prescribed analgesics, such as dextropropoxyphene or amitriptyline. Analgesic liniments were also used. A few of those with tension-type headache sometimes took dextropropoxyphene in addition to paracetamol.	24 h pattern of pain – needing medication at night
	Discontinuing medication, both prescribed and non-prescription analgesics, was reported by participants in all groups because of insufficient pain relief and side effects or fear of side effects.	

	Communicating with professionals and/or relatives and friends by asking, telling or complaining about their pain was described by almost all the participants. This concerned analgesics and/or sleeping aids or other treatments that they had heard or read about. They also expressed a desire for reduced physical therapy. The majority had spoken with, or complained to, various participants in their family, primarily their spouse, friends or caregivers, in an attempt to gain an understanding of their pain: "When I feel bad, I call a friend and talk with them. If you're really anger, I think it's a lot worse if you're in pain..." Making comparisons between before and after the stroke incident or between oneself and others who have suffered a stroke was described. The most common comparison made was with what the participants imagined could have happened to them, such as losing their ability to talk or becoming immobilized, which would then have been found to be harder to live with than the pain.	Side effects of medication<br><br>Social support - the need to communicate pain to try and gain understanding<br><br>Negative feeling – loneliness – lack of social support
	Distractions could take the form of various leisure activities, reading or sleeping to avoid experiencing the pain, or disciplining oneself to think positive in an attempt to forget the pain for a while. To the same end, the participants motivated themselves to get out and enjoy a change of scenery, or forgot their pain through nature experiences, hobbies, watching television, or listening to the radio or music of interest. This is illustrated in the following quote: "You have to get wrapped up in the disease as little as possible, I think. I read, and I listen to the radio from morning to night [laughs]. I have a smoke every now and then. I watch the news, nature programs, Jeopardy, and tennis. Sometimes I go to visit my daughter."	Positive thoughts<br><br>Distraction reduces pain – coping mechanism
	Rest and relaxation could involve resting from 10 to 30 minutes several times a day, in an attempt to get a chance of pain relief by stretching out in peace.	

Warming oneself by wearing warm clothing to avoid cooling down, which might induce pain, was reported by a few. To warm up by aquatic exercise was reported by those with central or nociceptive pain as an effort to relieve pain.	Reduces pain - easing factors - rest
Stopping activities was described by a few with nociceptive pain. They stopped whatever they were doing if they experienced pain in their shoulder or arm, or they no longer engaged in certain pain-provoking activities. This was also reported among those with tension-type headache, who would refrain from participating in a planned activity when they had headache.	Interventions to ease pain – keeping warm/ aquatic exercise<br>Aggravating factors - cold<br>Interventions to ease pain - rest/avoidance of provoking activity
Enduring the pain was described by participants in all groups since they could see no possibility of relief or cure, in spite of discomfort, uncertainty, and suffering for almost two years. This could mean gritting their teeth and fighting on, sometimes while continuing with their regular activities. Hopes for improvement were also expressed: "I'm waiting for it to get better, but I can live with the way things are now. I would be extremely grateful if it went away."	Acceptance of pain - continuing with activities despite the pain
Acceptance was expressed by a few participants, who said that they now had come to the point of accepting their pain and the problems that it brought with it, since they could not expect any improvement or relief. They said that they did not focus too much on the pain, but rather accepted it by living life in the here and now.	Hope
Experienced Outcomes of Coping The positive and negative experienced outcomes were in the narratives related to an effort to avoid or relieve the pain.	Acceptance of pain<br>Negative thoughts
Negative Outcome (Table 1). Perplexity and resignation were an experienced outcome, mainly as a result of the participants' efforts to communicate about their pain. Experienced lack of knowledge among	

	professionals and caregivers in instances where the participants had attempted to get answers, help and support were reported. Staff members had indicated they did not know what to do about the pain. The participants also found that they were not given consideration when they complained about pain in physical exercises during rehabilitation. Participants with central or nociceptive pain said that physicians had mainly told them that the pain would pass, but not when, or that "this is how it can be after a stroke," but not why. Those with tension-type headache had stopped asking questions because they had found that their problems were not being taken seriously. Accounts of experiences in which family members occasionally responded evasively or became irritated when the participants talked about their pain emerged in the narratives. Further, feelings of loneliness were described because of the participants' experiences of the family members' inability to provide support. Participants also related experiences in which they had not been believed because they had been thought of as lazy when they had attempted to avoid pain or because of their ability to look lively and happy. Some expressed disappointment at their inability to explain their pain in a way that others understood. Insufficient pain relief was experienced in connection with efforts to take both prescribed and non-prescription analgesics. Experienced frustration at seeing no possibility of being cured was prominent, although fear of or experienced side effects from the drugs and frustration over the absence of anticipated pain relief were related.	Incomprehensibility from HCPs<br><br>Incomprehensibility from relations<br><br>Lack of social support<br>Negative feelings – loneliness – lack of social support<br><br>Incomprehensibility from patients<br><br>Mood – frustration<br><br>Fear of side effects
**Widar, Ahlstrom and Ek, [35]**<br>Health-related quality of life in persons with long-term pain after a stroke	Physical aspects<br>Physical aspects were described in terms of freedom from pain and in terms of having one's health, in terms of retaining physical and cognitive capacity, and in terms of being physically independent. Freedom from pain was expressed in the following way:	

	More or less being able to live a good life without pain and… illness.	Improved QOL when free from pain, retaining physical and cognitive capacity and being physically independent.
	Physical and cognitive capacity emerged in the form of being able to walk and exercise, being orientated in terms of time and space and being able to communicate with others:<br>… that I can get up in the morning, can walk and can think… think clearly.<br>And then I think that in particular, that one still has the ability to speak means that one can talk to people, which of course means an awful lot.	Positive thoughts - having the physical ability to walk and exercise enhances cognitive capacity – orientated and able to communicate
	Physical independence emerged in the form of being able to take care of oneself and one’s daily life:<br>Yes, it is I suppose that I am able to be somewhat fit and stand on my legs and not need to be a vegetable, as they say, and to be spared from turning to others.	
	Psychological aspects<br>These concerned, principally, well-being, contentment, and time and freedom to be able to choose. Well-being emerged in the form of being able to retain self-esteem and being able to share a cheerful atmosphere with others:<br>That I am happy with myself, happy with my surroundings.<br>Contentment means being content with life despite altered living conditions:<br>… I would have liked to retain the ability to move, but I’m not down in the dumps about that, instead you have to be satisfied with things as they are.<br>Among the old-age pensioners, freedom with regard to time and choice of activities and living conditions emerged as an important component of quality of life:<br>Oh yes, it is of course this freedom of being able to do everything you wish to do when you wish to do it.<br>Yes, being able to continue to live in your own home.	

Occupation	
Occupation meant, for some, the possibility of getting a change in their daily life, having paid employment outside the home and being occupied. Among the people in paid employment, it emerged that employment is meaningful:	Financial stability
Yes it actually means a lot to go to work on weekdays and, quite simply, to work.	Routine/purpose
Leisure time meant being engaged in different kinds of leisure activities and which includes having the capacity for, possibility of and interest in the enjoyment of nature, society and club activities or a hobby, as the following remarks indicate:	
We go out, gladly go out into the forest every day.	
And then I have my book-binding circle when I can go to that, I consider that to be quality of life.	
Social and economic aspects	
These included the family and the home, i.e. experienced comfort and support from the family, their considerateness and practical care, as well as having the ability and possibility of being together.	Social support
Moreover, several persons considered that a sufficiently good financial situation was important as security, and not having to worry about that part in order to obtain variety in their situation. Under social aspects, the most prominent were family and relationships that work:	Financial stability
I have loving children and friends… a good home life.	
And then I have, of course, good kids who come and drive me around if I need it. Yes, and good neighbours and everything.	
Another important social aspect that emerged was social intercourse, the ability and possibility of being together with family and friends:	
And then that you are able to meet your children and grandchildren and other people.	
And that you have good friends. To be together with.	

Under economic aspects, economic security emerged as an important part of QOL in several persons:<br>That you are not without money but that you have enough to treat yourself. Yes, to be able to travel somewhere.
